# Supplementary material for: Towards unified AI-driven fracture mechanics: the extended deep energy method (XDEM)
Source: Nat Commun. 2026 Aug 15;17:8492. doi: 10.1038/s41467-026-76748-1 (PMC13484471; doi:10.1038/s41467-026-76748-1)
Supplement: Supplementary file 3 [file 41467_2026_76748_MOESM3_ESM.zip › pic/Results/Stress_intensity_factor/Cross/comparison_xdem_fem/angle_45/fem_dem_error_uv_3x2.pdf]

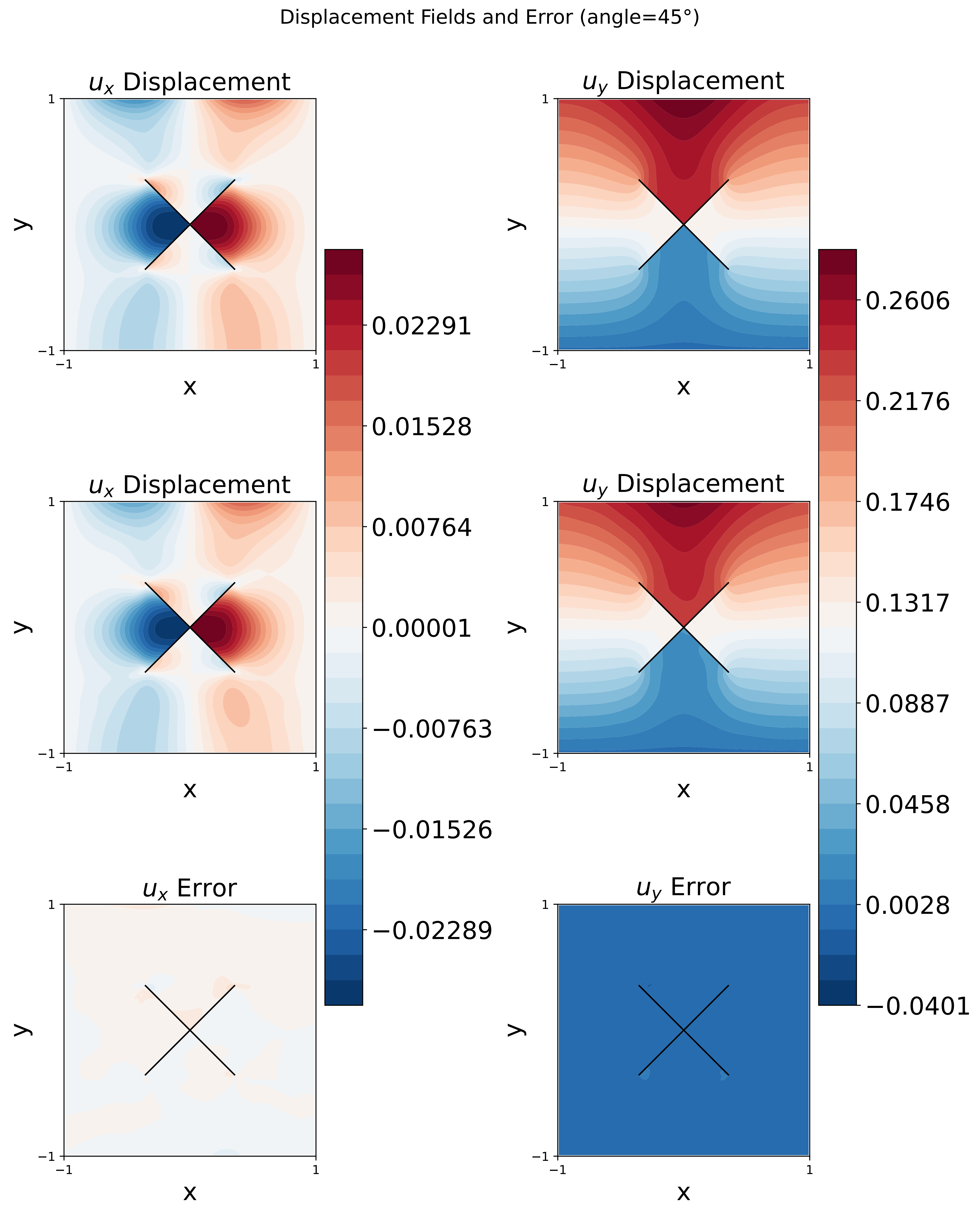

Displacement Fields and Error (angle=45°)
u_x Displacement
u_y Displacement
u_x Displacement
u_y Displacement
u_x Error
u_y Error
x
y
1
−1
0.02291
0.01528
0.00764
0.00001
−0.00763
−0.01526
−0.02289
0.2606
0.2176
0.1746
0.1317
0.0887
0.0458
0.0028
−0.0401
